# Shared genetic architecture of COVID-19 and Alzheimer’s disease

**DOI:** 10.3389/fnagi.2023.1287322

**Published:** 2023-10-20

**Authors:** Natalia Matveeva, Ivan Kiselev, Natalia Baulina, Ekaterina Semina, Viktor Kakotkin, Mikhail Agapov, Olga Kulakova, Olga Favorova

**Affiliations:** ^1^Institute of Medicine and Life Science, Immanuel Kant Baltic Federal University, Kaliningrad, Russia; ^2^Laboratory of Medical Genomics, Institute of Translational Medicine, Pirogov Russian National Research Medical University, Moscow, Russia

**Keywords:** COVID-19, long Covid, Alzheimer’s disease, cognitive impairment, pathway analysis, genetic susceptibility, GWAS, SNP

## Abstract

The severe acute respiratory syndrome-related coronavirus 2 (SARS-CoV-2) and the сoronavirus disease 2019 (COVID-19) have become a global health threat. At the height of the pandemic, major efforts were focused on reducing COVID-19-associated morbidity and mortality. Now is the time to study the long-term effects of the pandemic, particularly cognitive impairment associated with long COVID. In recent years much attention has been paid to the possible relationship between COVID-19 and Alzheimer’s disease, which is considered a main cause of age-related cognitive impairment. Genetic predisposition was shown for both COVID-19 and Alzheimer’s disease. However, the analysis of the similarity of the genetic architecture of these diseases is usually limited to indicating a positive genetic correlation between them. In this review, we have described intrinsic linkages between COVID-19 and Alzheimer’s disease, pointed out shared susceptibility genes that were previously identified in genome-wide association studies of both COVID-19 and Alzheimer’s disease, and highlighted a panel of SNPs that includes candidate genetic risk markers of the long COVID-associated cognitive impairment.

## 1. Introduction

In recent years the SARS-CoV-2 and the COVID-19 have become a global health threat. The most common COVID-19 symptoms are nonspecific and occur in many other infectious diseases. The course of COVID-19 can vary from mild to severe, up to death (Umakanthan et al., 2020); as of July 19, 2023, there are 6,951,677 COVID-19 deaths worldwide according to WHO data^1^. The severity of COVID-19 is affected by age and the presence of comorbidities like oncology or diseases of the cardiovascular system, lungs, kidneys, etc. (Wang et al., 2020).

In a significant proportion of patients (up to 20%), COVID-19 develops into a long-term pathological condition characterized by symptoms lasting more than 12 weeks and initially called the post-COVID-19 syndrome. Now both the post-COVID-19 syndrome and ongoing symptomatic COVID-19 lasting 4 to 12 weeks are combined into another term, long COVID, which is increasingly being used in the literature (Greenhalgh et al., 2022). Clinical manifestations of the long COVID are varied and often include fatigue, breathlessness, impaired blood clotting, persistent immunosuppression, headache, chest pain, muscle and joint pain, impaired sense of smell and taste (Yong, 2021; Oronsky et al., 2023), as well as various neuropsychiatric symptoms including cognitive impairment (Yong, 2021; Efstathiou et al., 2022). Surprisingly, the development of the long COVID does not depend on the severity of COVID-19 itself; it has been described even in patients with mild or asymptomatic disease (Yong, 2021). The long COVID is multifaceted, it usually affects multiple systems or organs and results in dramatic decline in quality of life and increased mortality. At the height of the pandemic, major efforts were focused on reducing COVID-19-associated morbidity and mortality. Now is the time to study the long-term effects of the pandemic, particularly cognitive impairment in the long COVID.

## 2. Cognitive impairment in the long COVID

The relationship between COVID-19 and cognitive impairment encompasses two main aspects: the first is the peculiarities of the course of COVID-19 in patients with psychiatric and neurodegenerative conditions and the second is a long COVID-associated cognitive impairment, which depends on the age of the patient (Herrera and González-Nosti, 2022). It has been convincingly shown that the infection affects the age of the first onset of various neurodegenerative diseases and their rate of progression (Rahmati et al., 2023). In the long term, the aspect of the long COVID-associated cognitive impairment is of particular importance, and we will focus on it in this review.

The main manifestations of cognitive dysfunction after COVID-19 are fatigue, “brain fog,” memory problems, attention deficit, sleep disturbances, anxiety, and depression (Premraj et al., 2022). Even 4 months after hospitalization, 30–40% of COVID-19 patients experience memory, attention, and sleep problems (Garrigues et al., 2020). Meta-analysis data show that patients who have recovered from COVID-19 experience a decline in overall cognitive abilities, when compared to healthy individuals, for a minimum of 7 months following the COVID-19 diagnosis (Crivelli et al., 2022). Even young adults who experienced a mild course of COVID-19, are at risk of developing cognitive symptoms (Gordon et al., 2022). Moreover, the mean age of patients who developed cognitive impairment after COVID-19 is significantly lower compared to those who did not (46.65 ± 9.08 vs. 50.05 ± 9.05, *p* < 0.001) (Matias-Guiu et al., 2023). The aforementioned facts emphasize high social significance of the long COVID and associated cognitive impairment.

## 3. Links between COVID-19 and Alzheimer’s disease

Alzheimer’s disease (AD) is a severe neurodegenerative disorder of the central nervous system (CNS) characterized by irreversible and progressive loss of cognitive function due to metabolic dysfunction, accumulation of misfolded proteins – beta-amyloid and tau protein – in brain tissue, and progressive neuronal death (Kunkle et al., 2019). AD is considered the primary cause of cognitive impairment in older individuals, responsible for over two-thirds of all dementia cases in people over 65 years of age (Kumar et al., 2023).

COVID-19 may provoke the development of AD. Within 360 days following a COVID-19 diagnosis, individuals aged 65 and above exhibited a significantly increased risk of the first diagnosis of AD (Wang et al., 2022a). In several Mendelian randomized studies, hospitalization with COVID-19 has been shown to be significantly associated with an increased risk of developing AD (Li et al., 2022; Baranova et al., 2023). The topic of interactions and intrinsic linkages between COVID-19 and AD is the subject of reviews summarizing data up to 2023 (Axenhus and Winblad, 2023; Li et al., 2023).

Recently, the similarity of the long COVID and AD has attracted increasing attention. It was noted that the consequences of COVID-19 may resemble the decline of cognitive functions commonly seen in aging (Mavrikaki et al., 2022). Moreover, after COVID-19, pathologic changes in the CNS similar to certain changes in AD are detected. It has been shown that among hospitalized COVID-19 patients, levels of biomarkers associated with neurodegeneration (such as beta-amyloid protein, tau protein, glial fibrillary acidic protein, neurofilament light chain, and ubiquitin carboxy-terminal hydrolase L1), increase to levels observed in AD-induced dementia and correlate with older age and severity of COVID illness (Frontera et al., 2022).

Рossible mechanisms underlying neurological consequences of COVID-19 have been summarized in a number of reviews (Jha et al., 2021; Mannekote Thippaiah et al., 2023). It has been established that SARS-CoV-2 can penetrate CNS (Pezzini and Padovani, 2020). Upon neuroinvasion, the SARS-CoV-2 spike protein binds to angiotensin-converting enzyme 2 (ACE2) on capillary endothelium, which acts as a receptor for viral entry into the cell A systemic exacerbated release of pro-inflammatory cytokines (so-called “cytokine storm”), characteristic of SARS-CoV-2, leads to increase in blood–brain barrier (BBB) permeability and facilitates virus penetration into the CNS. Another way for the virus to enter brain tissues is through the cells of the olfactory epithelium and olfactory tract (Baig et al., 2020). In the CNS, SARS-CoV-2 can interact with neurons and glial cells, which highly express ACE2 (Jha et al., 2021) and trigger a neuroinflammatory cascade, including activation of microglia, local production of proinflammatory cytokines, activation of inflammatory signaling, and subsequent astroglial reactivity, all of which may lead to neurodegeneration (Steardo et al., 2020; Saucier et al., 2023).

ACE2 is a key player in maintaining the balance between the pressor axis and the tissue-protective axis of renin-angiotensin system (RAS). ACE2 converts Angiotensin II (Ang II) to an active heptopeptide Ang-(1–7) which induces vasorelaxation, suppresses inflammation, oxidative stress, apoptosis, fibrosis and coagulation [summarized in (Heyman et al., 2021a)]. Binding of the receptor-binding domain (RBD) of the SARS-CoV-2 spike protein to ACE2 results in the internalization of the complex and depletion of ACE2 on the cell membrane. As a result, Ang-(1–7) production decreases and the activity of the pressor axis, potentiated by Ang II, rises. This eventually leads to inflammation, oxidative stress, tissue damage and coagulopathy typical for COVID-19 (Heyman et al., 2021b). In the CNS, the weakening of the protective RAS axis upon virus binding with ACE2 makes the neural tissue more vulnerable to damage (Baig et al., 2020). ACE2 depletion can cause an elevation in blood pressure and an increased risk of cerebral hemorrhage (Wu et al., 2020). Finally, ACE2 is involved in the beta-amyloid metabolism providing an additional link between COVID-19 and AD (Evans et al., 2020). Based on the body of experimental data reviewed, Reiss et al. distinguished three main mechanisms of the long-term neuropathological effects of SARS-CoV-2: (1) systemic inflammation leading to weakening of the blood–brain barrier (BBB), cytokine entry into the CNS, microglial activation, and production of neuroinflammatory mediators; (2) direct viral invasion of microvascular endothelium of the BBB and impairment of the blood flow in the brain; (3) entry of viral particles into the brain via the nasal epithelium and olfactory bulb, what causes neurotoxicity and neuronal loss (Reiss et al., 2023). Each of these mechanisms can impair neuronal function and thus cognition.

## 4. Shared GWAS-identified genes, involved in the pathogenesis of COVID-19 and Alzheimer’s disease

For both COVID-19 and different neurodegenerative diseases, numerous genome-wide association studies (GWASs) have been conducted, providing evidence of a genetic predisposition to these conditions. However, in only a few works genome-wide data were analyzed in order to search for shared genes involved in the pathogenesis of both COVID-19 and AD. Direct comparison of two GWASs allowed to identify locus chr19:44,744,108–46,102,697 containing *APOE* gene as a possible risk region for both COVID-19 and AD (Qiu et al., 2022). In the work (Baranova et al., 2023), a broader search for possible shared genes was carried out using GWAS catalog data. However, the database was accessed not later than 2022.05.20, when some important COVID-19 GWASs and meta-analyses had not yet been published. At that time, only for two genes, *LUZP2* and *RBFOX1*, both COVID-19- and AD-associated SNPs were found at the genome-wide significance level *p* ≤ 5 × 10^−8^. For SNPs in 17 more genes (*ANO3*, *CCDC171*, *CSMD1*, *DAB1*, *ECHDC3*, *EDAR*, *FAT1*, *GLIS3*, *GRIN2B*, *NAALADL2*, *NKAIN2*, *NTM*, *RBMS3*, *SHANK2*, *ST18*, *TCF7L2*, and *UNC5D*) the association with COVID-19 and/or AD was observed on a significance level reaching only *p* ≤ 1 × 10^−5^. The *APOE* gene was not highlighted at all in this study.

In this study, we conducted a search for shared susceptibility genes for COVID-19 and AD in the GWAS Catalog database 13 months later than in Baranova et al. (2023), when the amount of information available in the database has significantly increased, especially for COVID-19. At the time we accessed the GWAS catalog, it contained 249 studies and 4,064 associations of SNPs with COVID-19 (*p* ≤ 1 × 10^−5^); for AD, 2588 associations at the same level of significance were found in 150 studies. Out of them, the number of SNPs that meet the genome-wide significance level (*p* ≤ 5 × 10^−8^) turned out to be 1,100 for COVID-19 and 1,550 for AD. By analyzing the entire dataset, we identified 39 protein-coding genes and seven non-protein-coding genes, which showed an association with both COVID-19 and AD; the list of these genes is presented in Table 1.

**Table 1:** Shared genes that carry one or the other SNP associated with both COVID-19 and Alzheimer’s disease at a genome-wide significance level (*p* ≤ 5 × 10^−8^).

Genomic region	Gene, associated with both COVID-19 and Alzheimer’s disease	Gene description	Reference to the most relevant GWAS, which shows the association of the gene with the disease	
			COVID-19	Alzheimer’s disease
1. Protein-coding genes involved in nervous system development and functioning				
1q32.1	*LRRN2*	Leucine rich repeat neuronal 2	Słomian et al. (2023)	Kulminski et al. (2022)
2p16.1	*BCL11A*	BAF chromatin remodeling complex subunit BCL11A	Pairo-Castineira et al. (2023)	Kulminski et al. (2022)
10q26.13	*TACC2*	Transforming acidic coiled-coil containing protein 2	Chung et al. (2022b)	Gouveia et al. (2022)
11q14.1	*DLG2*	Discs large MAGUK scaffold protein 2	Słomian et al. (2023)	Gouveia et al. (2022)
11q21	*MTMR2*	Myotubularin related protein 2	Słomian et al. (2023)	Kulminski et al. (2022)
11p14.3	*LUZP2*	Leucine zipper protein 2	Słomian et al. (2023)	Gouveia et al. (2022)
16p13.3	*RBFOX1*	RNA binding fox-1 homolog 1	Słomian et al. (2023)	Herold et al. (2016)
17q21.31	*CRHR1*	Corticotropin releasing hormone receptor 1	Yao et al. (2023)	Gouveia et al. (2022)
17q21.31	*MAPT*	Microtubule associated protein tau	Wang et al. (2022b)	Kulminski et al. (2022)
18q21.2	*DCC*	DCC netrin 1 receptor	Słomian et al. (2023)	Kulminski et al. (2022)
19p13.13	*CACNA1A*	Calcium voltage-gated channel subunit alpha1 A	Słomian et al. (2023)	Kang et al. (2021)
2. Protein-coding genes involved in immune and inflammatory response				
1q21.3	*IL6R*	Interleukin 6 receptor	Guo et al. (2022)	Kauwe et al. (2014)
2q37.1	*INPP5D*	Inositol polyphosphate-5-phosphatase D	Słomian et al. (2023)	Lambert et al. (2013)
3p21.31	*CCR3*	C-C motif chemokine receptor 3	Yao et al. (2023)	Kauwe et al. (2014)
6p21.32	*HLA-DQA1*	Major histocompatibility complex, class II, DQ alpha 1	Yao et al. (2023)	Jansen et al. (2019)
6p21.32	*HLA-DRB1*	Major histocompatibility complex, class II, DR beta 1	Yao et al. (2023)	Jansen et al. (2019)
7p21.3	*PHF14*	PHD finger protein 14	Słomian et al. (2023)	Herold et al. (2016)
10q26.13	*PLEKHA1*	Pleckstrin homology domain containing A1	Chung et al. (2022b)	Bellenguez et al. (2022)
16p11.2	*ITGAX*	Integrin subunit alpha X	Yao et al. (2023)	Gouveia et al. (2022)
19q13.32	*APOE*	Apolipoprotein E	Chung et al. (2022b)	Bellenguez et al. (2022)
19p13.3	*CNN2*	Calponin 2	Chung et al. (2022b)	Jansen et al. (2019)
3. Other protein-coding genes				
2p15	*WDPCP*	WD repeat containing planar cell polarity effector	Słomian et al. (2023)	Gouveia et al. (2022)
5q23.3	*CHSY3*	Chondroitin sulfate synthase 3	Słomian et al. (2023)	Gouveia et al. (2022)
6q25.1	*MTHFD1L*	Methylenetetrahydrofolate dehydrogenase NADP+ dependent 1 like	Słomian et al. (2023)	Naj et al. (2010)
6p22.2	*ABT1*	Activator of basal transcription 1	Yao et al. (2023)	Gouveia et al. (2022)
6p22.2	*HMGN4*	High mobility group nucleosomal binding domain 4	Yao et al. (2023)	Gouveia et al. (2022)
7p14.3	*CHN2*	Chimerin 2	Słomian et al. (2023)	Gouveia et al. (2022)
8q24.3	*DENND3*	DENN domain containing 3	Słomian et al. (2023)	Chung et al. (2022a)
8p22	*PSD3*	Pleckstrin and Sec7 domain containing 3	Słomian et al. (2023)	Gouveia et al. (2022)
11q21	*CEP57*	Centrosomal protein 57	Słomian et al. (2023)	Kulminski et al. (2022)
12q24.32	*TMEM132C*	Transmembrane protein 132C	Słomian et al. (2023)	Herold et al. (2016)
14q22.1	*FERMT2*	FERM domain containing kindlin 2	Słomian et al. (2023)	Gouveia et al. (2022)
14q32.12	*SLC24A4*	Solute carrier family 24 member 4	Słomian et al. (2023)	Gouveia et al. (2022)
15q21.3	*ALDH1A2*	Aldehyde dehydrogenase 1 family member A2	Chung et al. (2022b)	Zhu et al. (2019)
15q24.1	*CCDC33*	Coiled-coil domain containing 33	Słomian et al. (2023)	Gouveia et al. (2022)
16q23.1	*WWOX*	WW domain containing oxidoreductase	Słomian et al. (2023)	Gouveia et al. (2022)
17q21.31	*KANSL1*	KAT8 regulatory NSL complex subunit 1	Kousathanas et al. (2022)	Gouveia et al. (2022)
19q13.32	*TOMM40*	Translocase of outer mitochondrial membrane 40	Słomian et al. (2023)	Gouveia et al. (2022)
19q13.33	*FUT2*	Fucosyltransferase 2	Yao et al. (2023)	Wightman et al. (2021)
4. Non-protein-coding RNA genes				
2p16.1	*REL-DT LINC01185*	REL divergent transcript	Yao et al. (2023)	Herold et al. (2016)
5q14.3	*LINC00461*	Long intergenic non-protein coding RNA 461	Guo et al. (2022)	Kulminski et al. (2022)
5q14.3	*TMEM161B-DT*	TMEM161B divergent transcript	Guo et al. (2022)	Kulminski et al. (2022)
6p21.32	*TSBP1-AS1*	TSBP1 and BTNL2 antisense RNA 1	Słomian et al. (2023)	Adewuyi et al. (2022)
8q24.21	*CASC8*	Cancer susceptibility 8	Słomian et al. (2023)	Gouveia et al. (2022)
15q11.2	*PWRN1*	Prader-Willi region non-protein coding RNA 1	Słomian et al. (2023)	Gouveia et al. (2022)
17q21.31	*LINC02210*	Long intergenic non-protein coding RNA 2210	Yao et al. (2023)	Gouveia et al. (2022)

Since neuronal dysfunction and inflammation have a critical role in the development of cognitive impairment associated with both COVID-19 (Gholami et al., 2021) and AD (Forloni and Balducci, 2018), we, using data from GeneCards database (Stelzer et al., 2016), identified two large subgroups of protein-coding genes from Table 1: the first subgroup includes genes involved in nervous system development and functioning and the second subgroup includes genes involved in immune and inflammatory response. Functions of these genes are discussed in more detail below.

The cognitive impairment in AD is known to be based on the dysfunction of lipid metabolism and metabolism of amyloid beta and tau protein, which eventually lead to accumulation of protein aggregates (Kunkle et al., 2019). Among the shared genes, belonging to the first subgroup, we found *MAPT* encoding precursor of the tau protein (Tabeshmehr and Eftekharpour, 2023), *CRHR1* mediating tau hyperphosphorylation (Lemche, 2018) and *CACNA1A* involved in neuronal cell death and toxicity of amyloid beta (Demuro et al., 2010). In addition, four genes belonging to the first subgroup: *DLG2*, *MTMR2*, *RBFOX1*, and *DCC*, are involved in synapse formation and signal transduction (Lee et al., 2010; Alkallas et al., 2017; Wong et al., 2019; Prokopenko et al., 2022), what may indicate deregulation of these processes in the development of COVID-19 and AD-associated cognitive impairment.

Functions of the several highlighted genes (*IL6R*, *INPP5D*, *CCR3*, *HLA-DQA1*, *HLA-DRB1*, *PHF14*, *PLEKHA1*, *ITGAX*, *APOE*, *CNN2*), belonging to the second subgroup of shared protein-coding genes, relate to the development of the immune response and inflammation, what is in a good agreement with the current understanding of the critical role of inflammatory responses in the development of various neurodegenerative diseases, particularly AD (Hammond et al., 2019). Many genes involved in the regulation of the immune response and inflammation have been found among the risk genes for such diseases (Nott and Holtman, 2023).

Microglial cells are key regulators of inflammatory responses in the central nervous system (Kwon and Koh, 2020), and studies at the genomic and transcriptomic levels showed that neurodegeneration may be strongly associated with altering microglia function (Raj et al., 2014; Lopes et al., 2022). Among the genes of the second subgroup there are several genes involved in the functioning of microglia and the maintenance of its homeostasis; they are associated with inflammation, cell adhesion and migration (*APOE*, *IL6R*, *CCR3*, *ITGAX*, *CNN2*), as well as with regulation of microglial gene expression (*INPP5D*). It has been established that the development of COVID-19 is also associated with various inflammatory reactions; in severe cases an abrupt and uncontrolled release of proinflammatory cytokines (“cytokine storm”) develops leading to damage to many cells and tissues (Hu et al., 2021). The association of the *APOE* gene with the development of both diseases has been most well studied (see section 5).

In addition to the genes associated with innate immune response, the second subgroup includes MHC class II genes *HLA-DQA1* and *HLA-DRB1* and some genes that regulate the formation of germinal centers and the production of autoantibodies (*PHF14* and *PLEKHA1*) (Sharma et al., 2018; Zhang et al., 2020), which suggests a possible involvement of adaptive immunity in cognitive impairment associated with both COVID-19 and AD.

According to the GeneCards database (Stelzer et al., 2016), functions of other protein-coding genes combined in the third subgroup relate to the chromosome organization and regulation of transcription (*ABT1*, *HMGN4*, *CEP57*, *KANSL1*), cell signaling (*CHSY3*, *ALDH1A2*, *TMEM132C*, *FUT2*, *SLC24A4*), metabolic processes (*CHSY3*, *ALDH1A2*, *TMEM132C*, *FUT2*), cytoskeleton organization and cell motility (*WDPCP*, *FERMT2*), vesicle-mediated transport (*DENND3*, *PSD3*) and mitochondria functioning (*TOMM40*, *MTHFD1L*).

Finally, non-protein-coding genes that make up the fourth subgroup remain largely unexplored, with only exceptions being the miRNA sponge *LINC00461*, involved in the regulation of *MAPK/ERK* and PI3K/AKT signaling pathways (Zhang et al., 2022), and imprinted *PWRN1*, which is located in the Prader-Willi syndrome region and associated with various cancers (Jiang et al., 2020).

## 5. Candidate polymorphic variants, which may be involved both in COVID-19 and Alzheimer’s disease genetic architecture

The genetic architecture of COVID-19 and AD seems to exhibit certain similarities not only at the level of common genes involved in the same pathological processes, but also at the level of shared or linked particular polymorphic variants, what is confirmed by a positive genetic correlation between the diseases (Li et al., 2022; Qiu et al., 2022; Baranova et al., 2023). To date the only genetic variant known to be associated with both COVID-19 and AD is ε4 allele of the *APOE* gene (Xiong et al., 2021).

The *APOE* gene has three main alleles, ε2, ε3, and ε4, defined by a combination of variants of two SNPs: rs429358 and rs7412; these alleles encode three isoforms of the gene protein product, apolipoprotein E (ApoE), which differ from each other by amino acids at positions 112 and 158: ApoE2 (cys112, cys158), ApoE3 (cys112, arg158) and ApoE4 (arg112, arg158) (Weisgraber et al., 1981). ApoE4 stands apart from other ApoE isoforms, because its primary sequence causes formation of a more compact spatial structure and leads to the protein instability (Dong et al., 1994). ApoE is involved in transport of cholesterol and some other lipids. In the CNS it is mainly produced by astrocytes and microglial cells and involved in neuronal lipid metabolism and regulation of beta amyloid production, modifying risk of AD (Wang et al., 2021b). ApoE isoforms may affect microglial activation and neuroinflammatory state of the brain (Kloske et al., 2021) and thus modulate pathogenesis of both COVID-19 and AD. ApoE2 and ApoE3 isoforms were shown to inhibit beta amyloid aggregation and neurotoxicity, whereas ApoE4 lacks neuroprotective activity and facilitates AD (Drouet et al., 2001). ApoE4 seems to be involved in the neurological effects of COVID-19 by increasing rates of SARS-CoV-2 entry into neuronal cells and stimulating their apoptosis under infection (Wang et al., 2021a). The *APOE* ε4 allele is one of the primary genetic risk factors of AD (Raulin et al., 2022). It was also shown to significantly increase the chance to test positive for COVID-19 (Kuo et al., 2020a). The risk of symptomatic COVID-19, severe COVID-19 and disease-associated mortality were also elevated in *APOE* ε4 carriers (Kuo et al., 2020a,b; Del Ser et al., 2021; Hubacek et al., 2021).

In order to search for a panel of SNPs that can be used as genetic markers of the risk of the long COVID-associated cognitive impairment, we compared the lists of SNPs associated with COVID-19 or AD (*p* ≤ 5 × 10^−8^) and located in 46 shared genes listed in Table 1. Expectedly, *APOE* SNP rs429358, which, in combination with rs7412, is responsible for the formation of three main *APOE* alleles, was associated with both COVID-19 and AD (highest value of ps extracted from GWAS catalog were equal to 1 × 10^−44^ and 2 × 10^−303^, respectively); no significant shared SNPs were found for other genes.

Next, for each gene presented in the Тable 1 we have chosen the pairs of SNPs, in which one SNP is associated with COVID-19 and the other with AD, and extracted from LDlink database (Machiela and Chanock, 2015) data on linkage disequilibrium between these SNPs for the general population (complete dataset, ALL) and for the most represented in GWASs European populations (EUR) of the 1,000 Genomes Project. Regions of genes *TSBP1-AS1*, *DLG2*, *PWRN1*, *CCDC33*, and *CACNA1A* were excluded from this analysis because some of their SNPs were either absent in the 1,000 Genomes reference panel, or are not biallelic. If several SNPs of the same gene were associated with COVID-19 and/or AD, only the pair characterized by the highest R^2^ value was selected. As a result we pinpointed 19 pairs of SNPs associated with COVID-19 and AD and characterized by significant linkage disequilibrium (Table 2). Among them, 3 pairs of SNPs (in *BCL11A*, *CHN2*, and *CNN2* genes) were in the linkage disequilibrium in European populations only. Three gene pairs (*LINC00461* and *TMEM161B-DT*, *HLA-DQA1*, and *HLA-DRB1*, *ABT1* and *HMGN4*) located in the same genomic loci were merged together in the Table 2 because they were assigned to the same SNPs. We believe that in addition to the *APOE* SNP rs429358, SNPs included in the Table 2 may be good candidate genetic risk markers of the long COVID-associated cognitive impairment. Unfortunately, the effects of alleles and genotypes of certain identified SNPs on the expression of genes, in which they are located, still remain unknown and require further research.

**Table 2:** SNPs in the shared genes, associated with COVID-19 and Alzheimer’s disease in GWASs, which are in linkage disequilibrium.

Gene(s)	SNP rsID (highest value of *p* obtained in GWASs)*		Linkage disequilibrium, D′ (R^2^) according to the LDlink database	
	COVID-19	Alzheimer’s disease	General population (ALL)	European samples only (EUR)
*IL6R*	rs6694817 (2.0 × 10^−10^)	rs61812598 (6 × 10^−63^)	0.96 (0.29)	0.96 (0.41)
*INPP5D*	rs6717453 (1 × 10^−15^)	rs35349669 (3 × 10^−8^)	0.76 (0.25)	0.76 (0.46)
*BCL11A*	rs1123573 (2 × 10^−14^)	rs7599488 (1 × 10^−11^)	Non-significant	0.45 (0.18)
*LINC00461, TMEM161B-DT*	rs17419291 (3 × 10^−13^)	rs7737179 (2 × 10^−8^)	0.96 (0.13)	0.94 (0.2546)
*HLA-DQA1, HLA-DRB1*	rs601945 (5 × 10^−567^)	rs6931277 (8 × 10^−11^)	0.98 (0.69)	0.99 (0.98)
*ABT1, HMGN4*	rs9379892 (3 × 10^−11^)	rs6925895 (1 × 10^−8^)	0.96 (0.19)	1.00 (0.25)
*CHN2*	rs10228072 (2 × 10^−11^)	rs10268281 (9 × 10^−9^)	Non-significant	0.43 (0.18)
*PLEKHA1*	rs11200629 (7 × 10^−221^)	rs7908662 (3 × 10^−9^)	1.00 (0.99)	1.00 (1.00)
*CEP57*	rs666398 (1 × 10^−19^)	rs535819 (5 × 10^−8^)	1.00 (0.66)	1.00 (0.81)
*MTMR2*	rs473157 (5 × 10^−13^)	rs3824874 (5 × 10^−9^)	0.97 (0.18)	0.97 (0.54)
*FERMT2*	rs12878516 (7 × 10^−11^)	rs17125944 (8 × 10^−9^)	0.95 (0.17)	0.98 (0.13)
*SLC24A4*	rs2402144 (2 × 10^−25^)	rs35627364 (2 × 10^−10^)	0.53 (0.14)	0.57 (0.21)
*ALDH1A2*	rs2414577 (3 × 10^−16^)	rs261291 (4 × 10^−84^)	0.80 (0.39)	0.97 (0.81)
*ITGAX*	rs61194152 (1 × 10^−8^)	rs7190997 (2 × 10^−12^)	0.50 (0.16)	0.37 (0.11)
*KANSL1*	rs2532300 (4 × 10^−9^)	rs2696697 (5 × 10^−16^)	1.00 (0.99)	1.00 (1.00)
*MAPT*	rs4792891 (7 × 10^−10^)	rs113568679 (7 × 10^−9^)	1.00 (0.33)	1.00 (0.55)
*TOMM40*	rs1038026 (2 × 10^−13^)	rs7259620 (3 × 10^−156^)	1.00 (0.77)	1.00 (1.00)
*FUT2*	rs492602 (4 × 10^−16^)	rs2452170 (2 × 10^−8^)	0.95 (0.86)	0.97 (0.84)
*CNN2*	rs67538026 (4 × 10^−9^)	rs111278892 (8 × 10^−11^)	Non-significant	0.84 (0.17)

*Corresponding references are shown in Table 1.

## 6. Conclusion

The assessment of the risk of developing cognitive impairment as a manifestation of the long COVID is of undoubted practical importance. Identification of high-risk groups among COVID-19 survivors may help to prevent cognitive impairment through a combination of lifestyle changes and medication interventions.

However, it is hindered due to a number of different factors. Only a small number of people undergo regular cognitive tests, which significantly limits the ability to assess cognitive impairment before and after COVID-19 (Nami et al., 2022). At the same time, the lack of unified protocols for the assessment of cognitive functions complicates the interpretation of the obtained data. In addition, hypoxia, endothelial dysfunction, intoxication, thrombosis, etc., which often accompany COVID-19, in some cases may act as nonspecific causes of cognitive impairment. And finally, the prolonged isolation, fear, and financial losses in COVID-19 can by themselves lead to depression, anxiety disorders, sleep disturbances, difficulties in concentration, and other symptoms of post-traumatic stress (Hossain et al., 2020).

Against the background of these modifiable factors, analysis of the genetic susceptibility may be a reliable method for predicting the risk of developing cognitive impairment due to COVID-19. Selection of the most informative risk markers among SNPs, which are localized in shared genes associated with COVID-19 and AD, assessment of their sensitivity and specificity for predicting the risk of developing cognitive impairment among COVID-19 survivors, and creation of an effective SNP panel to predict the individual risk is an urgent task of the near future.

## Author contributions

NM: Conceptualization, Writing – original draft. IK: Conceptualization, Writing – original draft. NB: Writing – review & editing. ES: Writing – review & editing. VK: Writing – review & editing. MA: Writing – review & editing. OK: Supervision, Writing – review & editing. OF: Funding acquisition, Project administration, Writing – review & editing.

## Footnotes

^1^ https://covid19.who.int/
